# Supplementary material for: UHRF2 mediates resistance to DNA methylation reprogramming in primordial germ cells
Source: Nat Commun. 2025 Aug 9;16:7350. doi: 10.1038/s41467-025-61954-0 (PMC12335541; doi:10.1038/s41467-025-61954-0)
Supplement: Supplementary file 1 — Supplementary Information [file 41467_2025_61954_MOESM1_ESM.pdf]

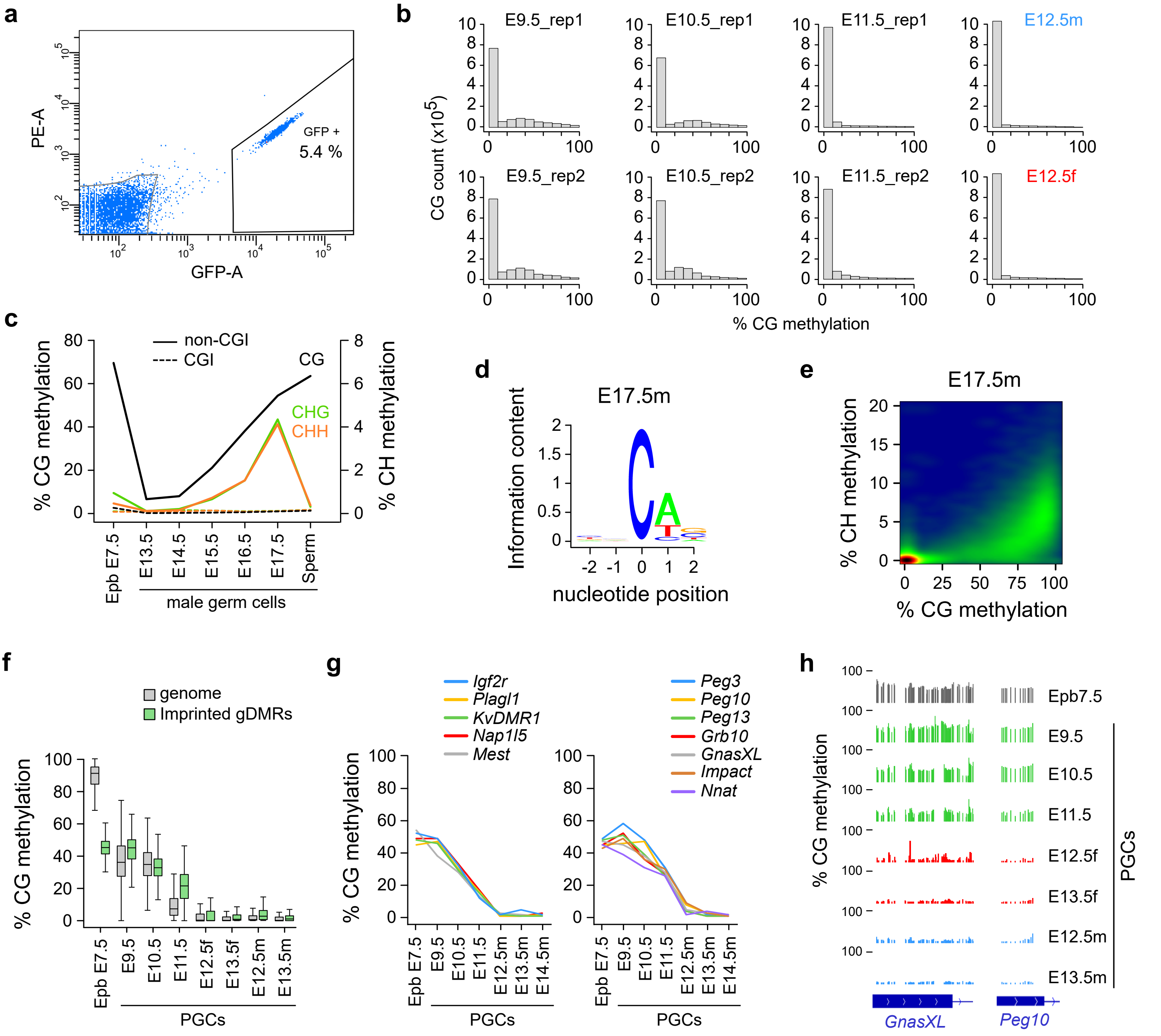

Figure S1. Detailed analysis of DNA methylation dynamics in PGCs. a) Representative example of FACS profile for the purification of PGCs from E13.5 gonads of embryos expressing the *Oct4*-GFP transgene. The cells were gated based on the GFP signal (x-axis) and GFP autofluorescence (PE-A, y-axis). **b)** Histograms showing the distribution of CG methylation by RRBS in two independent pools of E9.5-E11.5 PGCs, as well as male and female E12.5 PGCs. **c)** Dynamics of CG and CH methylation measured by RRBS in prospermatogonia and spermatozoa. In this graph, the values are shown separately for cytosines in CpG islands (CGI, dashed lines) and outside of CpG islands (non-CGI, full lines). **d)** Logo showing the sequence context of methylated (>2%) CH sites in E17.5 prospermatogonia. **e)** Pairwise correlation of CG and CH methylation levels in 500 bp windows in E17.5 prospermatogonia. **f)** Comparison of the kinetics of CG demethylation in imprinted germline DMRs (gDMRs) compared to the whole genome. For the genome, we selected all CGs with a methylation >50% in epiblast (Epb) and <20% in E13.5 PGCs. Boxplots: center line indicates the median, box limits indicate upper and lower quartiles, whiskers extend to 1.5 interquartile range. **g)** Kinetics of CG demethylatrion in several gDMRs of imprinted genes. **h)** Examples of RRBS methylation profiles in the *Gnas*XL and *Peg10* gDMRs during PGC reprogramming. f: female, m: male.

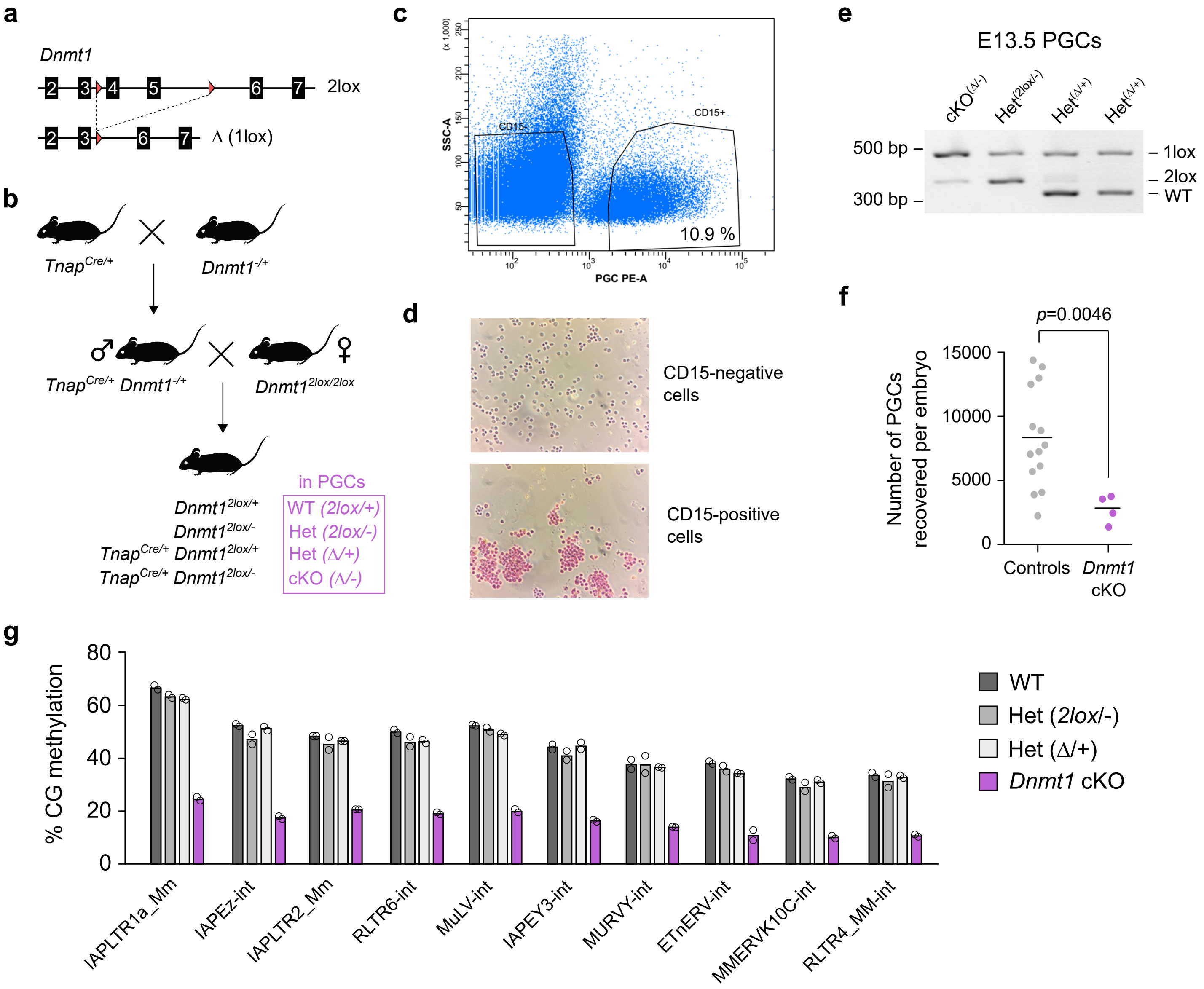

Figure S2. Generation and analysis of *Dnmt1* conditional KO mice. a) Schematic representation of the *Dnmt1* floxed allele (2lox) and knockout allele resulting from Cre-mediated recombination (1lox). The loxP sites are depicted by triangles. **b)** Mouse breeding scheme used to generate a conditional knockout of *Dnmt1* in PGCs with the *Tnap*-Cre line. **c)** Representative example of FACS profile for the purification of PGCs from E13.5 gonads using anti-SSEA1 (CD15) antibodies. **d)** Alkaline phosphatase staining of CD15-positive and CD15-negative cells recovered by FACS from E13.5 gonads. **e)** Representative example of PCR genotyping showing the Cre-mediated excision of *Dnmt1* exons in cKO PGCs. **f)** Number of PGCs recovered in *Dnmt1* cKO compared to control (WT and heterozygous littermates) E13.5 embryos (controls n=14, cKO n=4). The horizontal line represents the mean value. *p*-value: two-sided Mann-Withney test. **g)** Mean methylation levels of ERV families in E13.5 PGCS from *Dnmt1* cKO compared to control embryos (n=2 embryos per genotype).

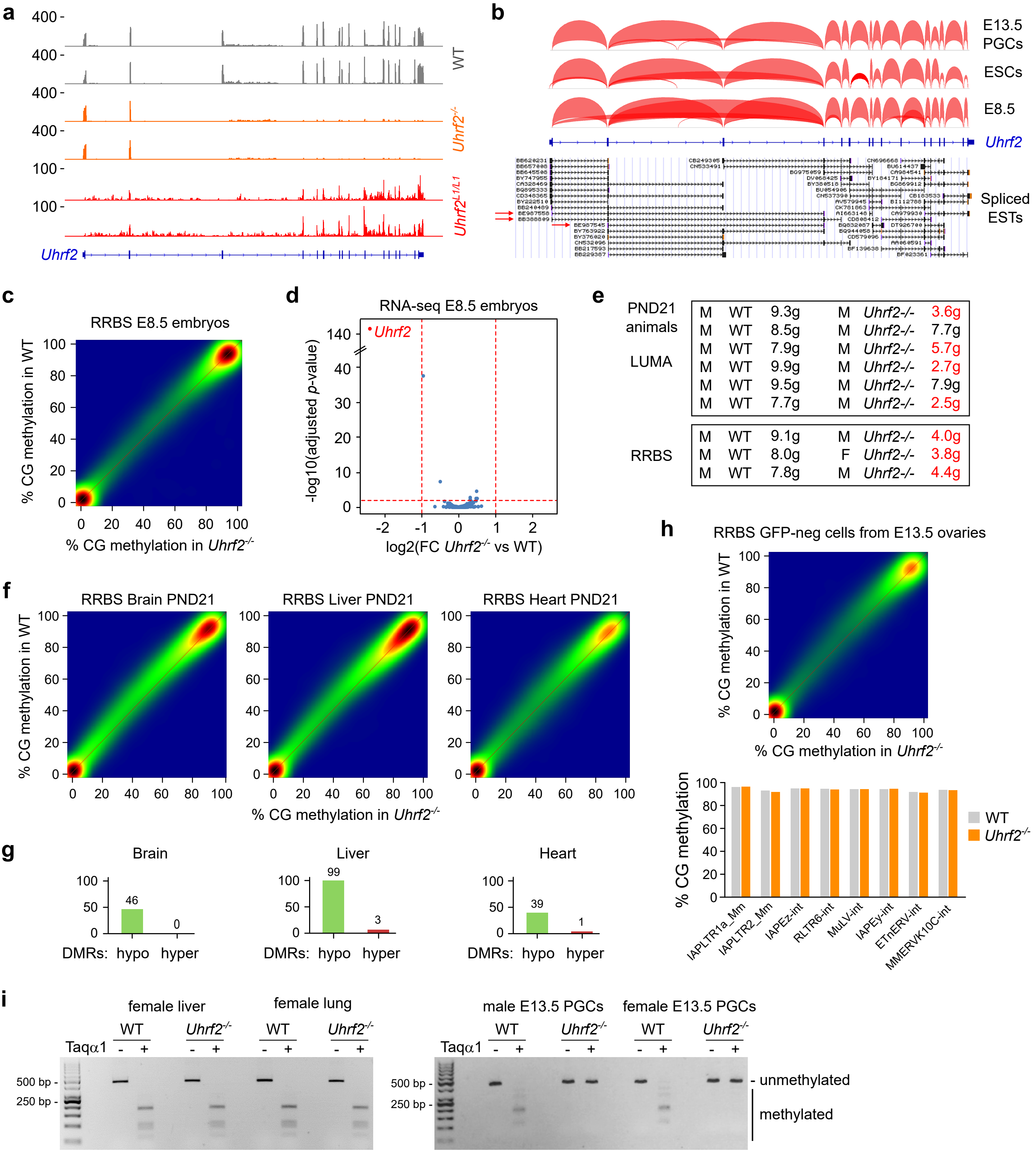

Figure S3. Additional analyses in *Uhrf2* deficient mice. a) *Uhrf2* RNA-seq tracks in WT and mutant E8.5 embryos. The exon 3 is absent in *Uhrf2*$^{L1/L1}$ embryos, whereas the expression of exon 3 and downstream exons is abolished in *Uhrf2*$^{-/-}$ animals. **b)** *Uhrf2* splice junction tracks in E13.5 PGCs, ESCs and E8.5 embryos. Spliced ESTs from the UCSC genome browser are shown below the RefSeq annotation. Red arrows highlight spliced ESTs skipping the exon 3. **c)** Pairwise correlation of RRBS CG methylation scores in 500 bp windows in *Uhrf2*$^{-/-}$ vs WT E8.5 embryos (average of n=2 embryos per genotype). **d)** Volcano plot representation of differential gene expression in *Uhrf2*$^{-/-}$ vs WT E8.5 embryos. Differentially expressed genes (FC >1, padj < 0.001) are shown in red. *P*-values: DESeq2 adjusted *p*-values. **e)** Body weight of PND21 animals used for DNA methylation analysis by LUMA or RRBS. Growth retarded animals are highlighted in red. **f)** Correlations of RRBS CG methylation scores in 500 bp windows in brain, liver and heart of *Uhrf2*$^{-/-}$ vs WT PND21 animals (average of n=2 or 3 independent animals per genotype). **g)** Number of DMRs identified by RRBS in brain, liver and heart of *Uhrf2*$^{-/-}$ PND21 animals. **h)** DNA methylation analysis by RRBS in GFP-negative cells from an *Uhrf2*$^{-/-}$ vs WT E13.5 ovary. Top: correlation of CG methylation scores in 500 bp windows; bottom: methylation of ERV families. **i)** COBRA of IAPEz in E13.5 PGCs and somatic cells of *Uhrf2*$^{-/-}$ and WT mice. The experiment was performed once.

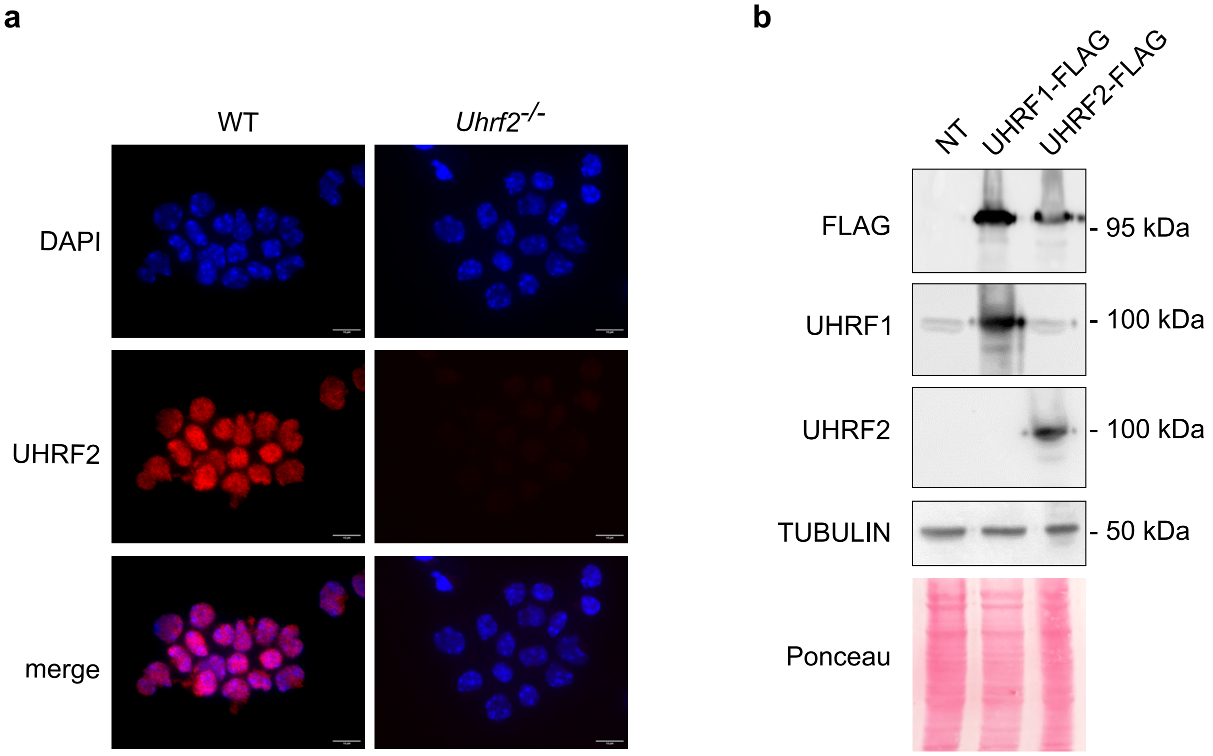

Figure S4. Specificity of the anti-UHRF1 and anti-UHRF2 antibodies. a) DAPI staining and immunofluorescence analysis with the anti-UHRF2 antibody in E13.5 gonadal cells from a WT and *Uhrf2*$^{-/-}$ female. The experiment was performed independently twice with similar results. Scale bar: 10 µM. **b)** Western blots with anti-FLAG, anti-UHRF1 and anti-UHRF2 antibodies on protein extracts from HEK293T cells transiently transfected with plasmids expressing FLAG-tagged mouse UHRF1 or UHRF2 compared to non-transfected (NT) cells. α-TUBULIN and Ponceau staining are shown as loading controls. The experiment was performed once.

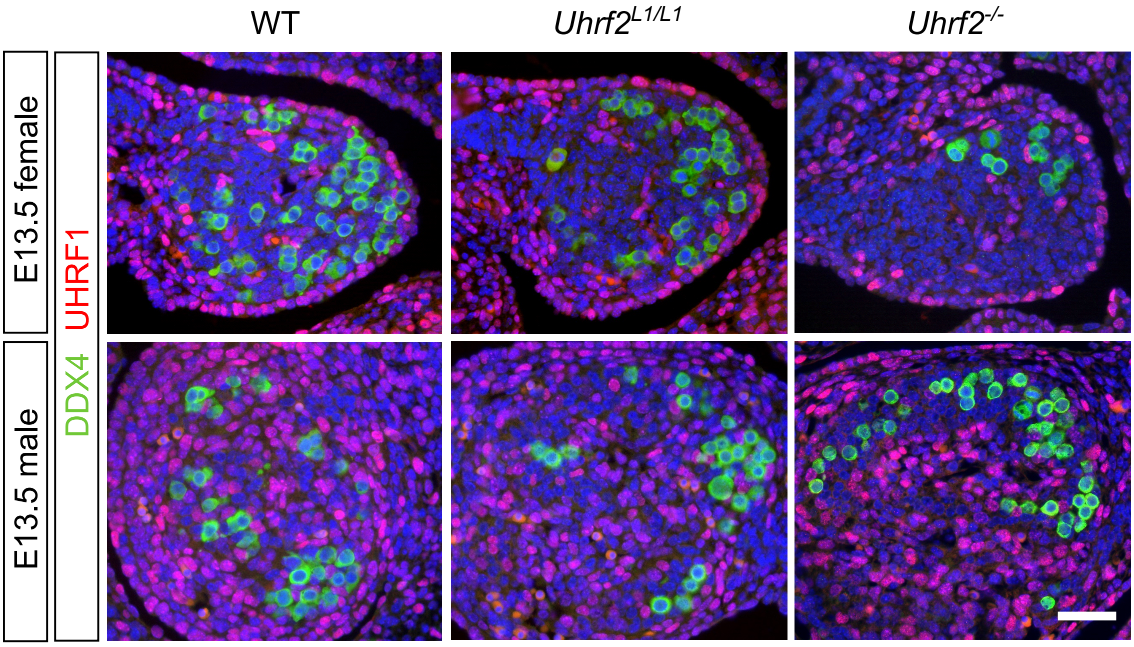

Figure S5. UHRF1 is not ectopically expressed in mutant PGCs lacking UHRF2. UHRF1 expression was analyzed by immunostaining (red nuclear signal) on sections of ovaries and testis from control (WT) and *Uhrf2* mutant E13.5 fetuses, as indicated. Immunostaining of DDX4 (green signal) was used to detect the PGCs. Nuclei are counterstained with DAPI (blue signal). The experiments were repeated independently at least on four gonads for each genotype. Scale bar: 30 µm.

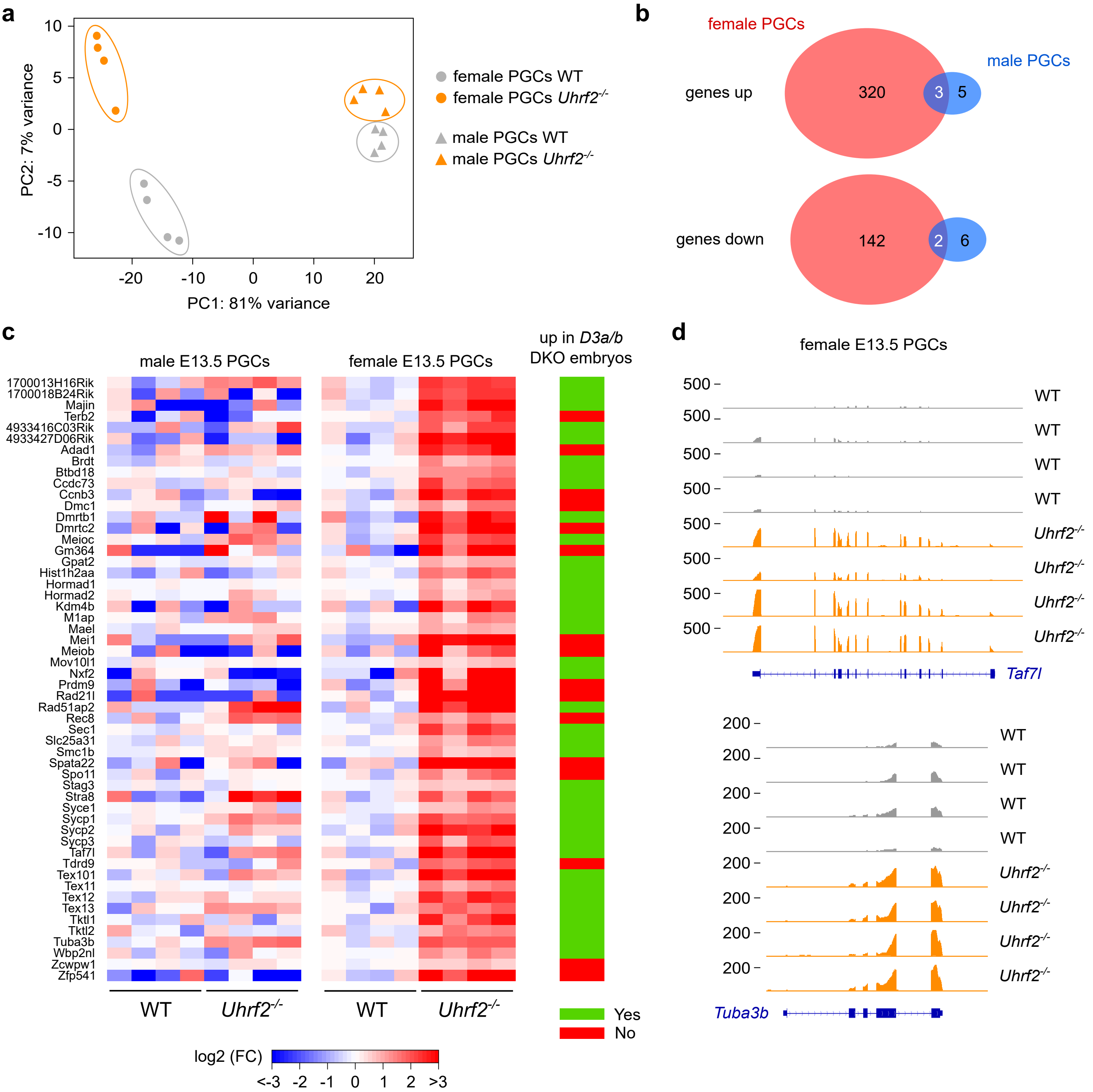

Figure S6. Transcriptome analysis by RNA-seq in *Uhrf2*$^{-/-}$ E13.5 PGCs. a) Principal Component Analysis of RNA-seq data in *Uhrf2*$^{-/-}$ and WT E13.5 PCGs (n=4 independent female and male embryos per genotype). **b)** Venn diagrams showing the overlap between genes significantly upregulated (top) and downregulated (bottom) in female and male *Uhrf2*$^{-/-}$ E13.5 PGCs. **c)** Heatmap of the expression of germline and meiotic genes overexpressed in female *Uhrf2*$^{-/-}$ E13.5 PGCs. The values are fold changes relative to the mean expression in WT PGCs of the same sex. The last column indicates whether the genes are among the list of significantly upregulated genes in *Dnmt3a/b* DKO mouse embryos (Dahlet et al., Nature Commun 2020). **d)** RNA-seq tracks for the *Taf7l* and *Tuba3b* genes in *Uhrf2*$^{-/-}$ compared to WT female E13.5 PGCs.

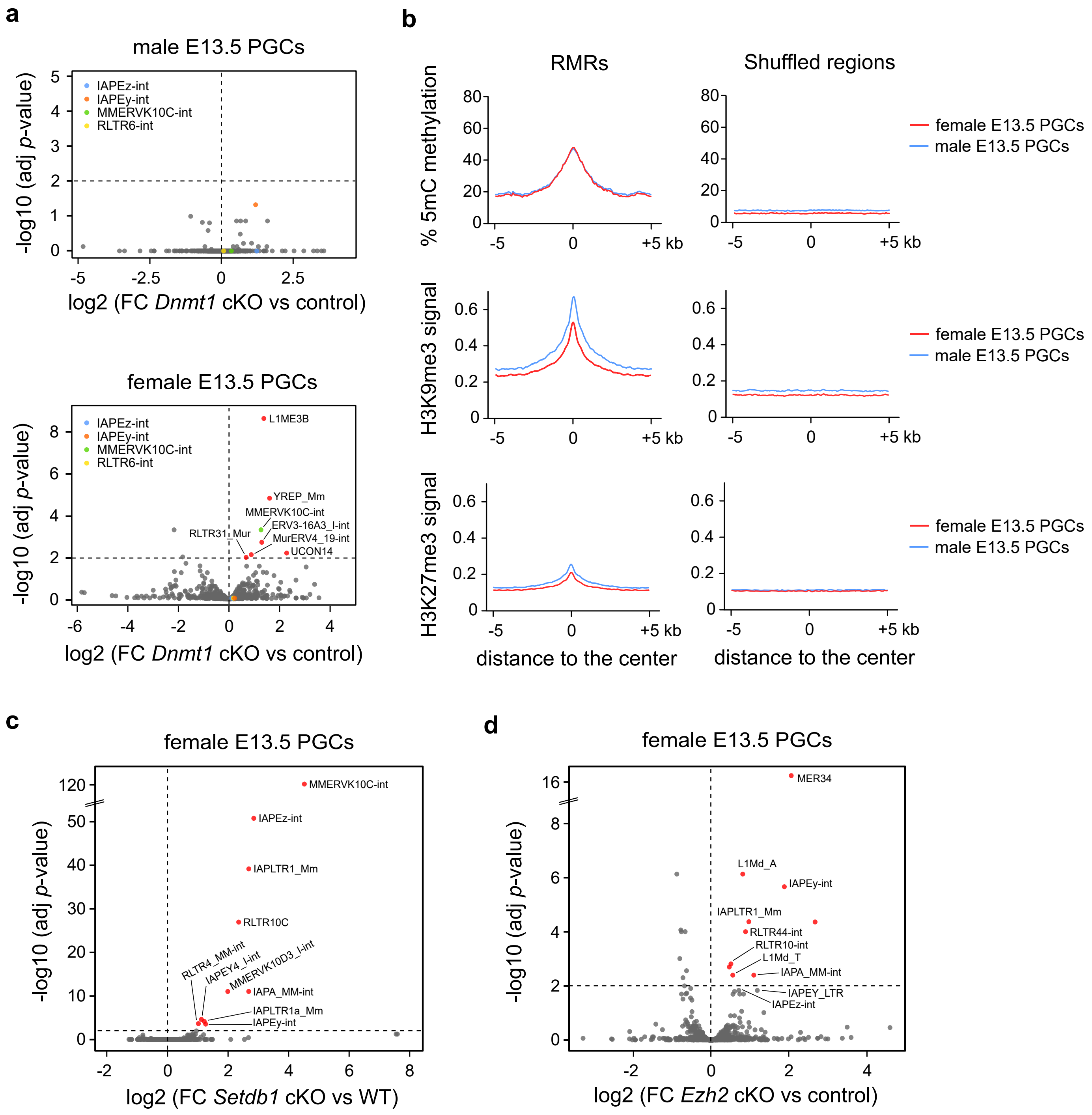

Figure S7. Marking and transcriptional control of RMRs by histone modifying pathways in PGCs. a) Volcano plots showing differential expression of TE families in male and female *Dnmt1* cKO E13.5 PGCs (reanalysis of RNA-seq data from Hargan-Calvopina et al., 2016). Significantly upregulated TE families (adjusted *p*-value< 0.01) are highlighted in red. *P*-values: DESeq2 adjusted *p*-values. **b)** Metaplots of H3K9me3 and H3K27me3 ChIP-seq signals over RMRs in E13.5 PGCs (reanalysis of ChIP-seq data from Liu et al., 2014). Metaplots for an equal number of randomly shuffled regions are shown as controls. **c)** Volcano plots showing differential expression of TE families in female *Setdb1* cKO E13.5 PGCs (reanalysis of RNA-seq data from Liu et al., 2014). Significantly upregulated TE families (adjusted *p*-value< 0.01) are highlighted in red. **d)** Volcano plots showing differential expression of TE families in female *Ezh2* cKO E13.5 PGCs (reanalysis of RNA-seq data from Huang et al., 2021). Significantly upregulated TE families (adjusted *p*-value< 0.01) are highlighted in red. *P*-values: DESeq2 adjusted *p*-values.

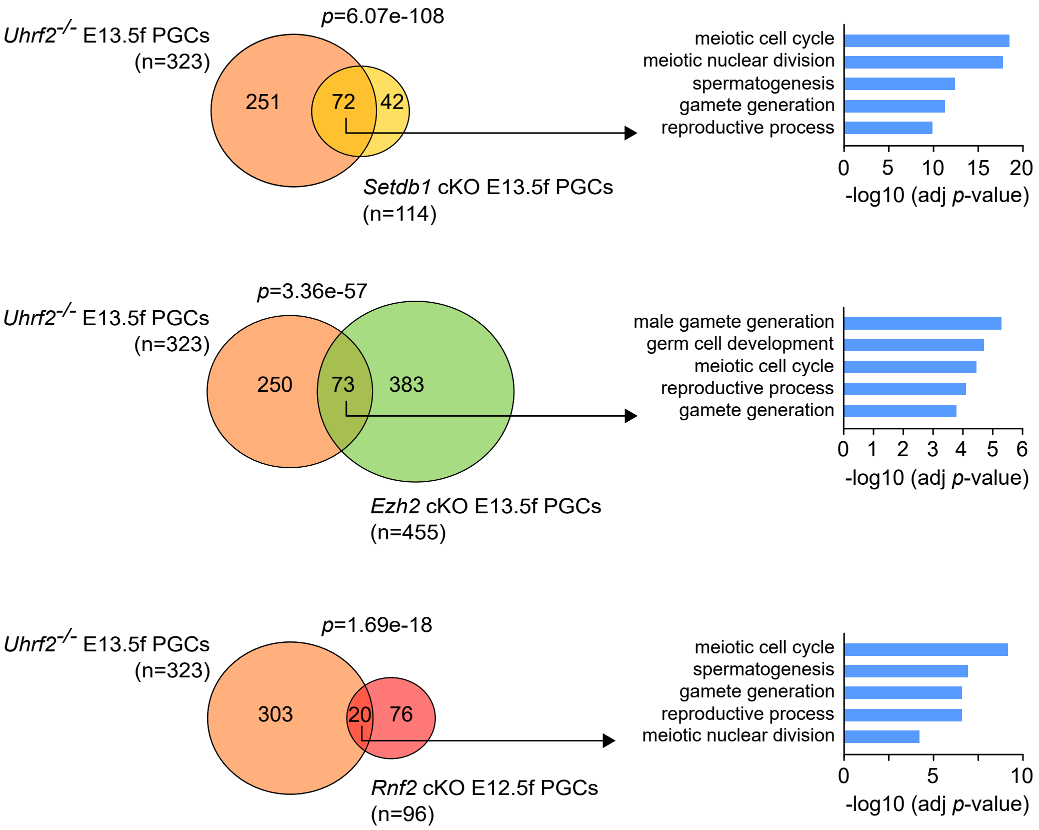

Figure S8. Comparison of the genes upregulated in *Uhrf2-/-* female PGCs and female PGCs mutant for other epigenetic pathways. The Venn diagrams show the overlap between the genes upregulated in *Uhrf2-/-* female (f) E13.5 PGCs (this study) and the genes upregulated in female E13.5 PGCs mutant for *Setdb1* (top, reanalysis fo RNA-seq data from Liu et al., 2014), female E13.5 PGCs mutant for *Ezh2* (middle, reanalysis fo RNA-seq data from Huang et al., 2021) and female E12.5 PGCs mutant for *Rnf2* (bottom, microarray data from Yokobayashi et al., 2013). *P*-values to measure the significance of the overlaps were calculated by hypergeometric tests. The graphs on the right show the gene ontology terms significantly enriched among the lists of common genes.

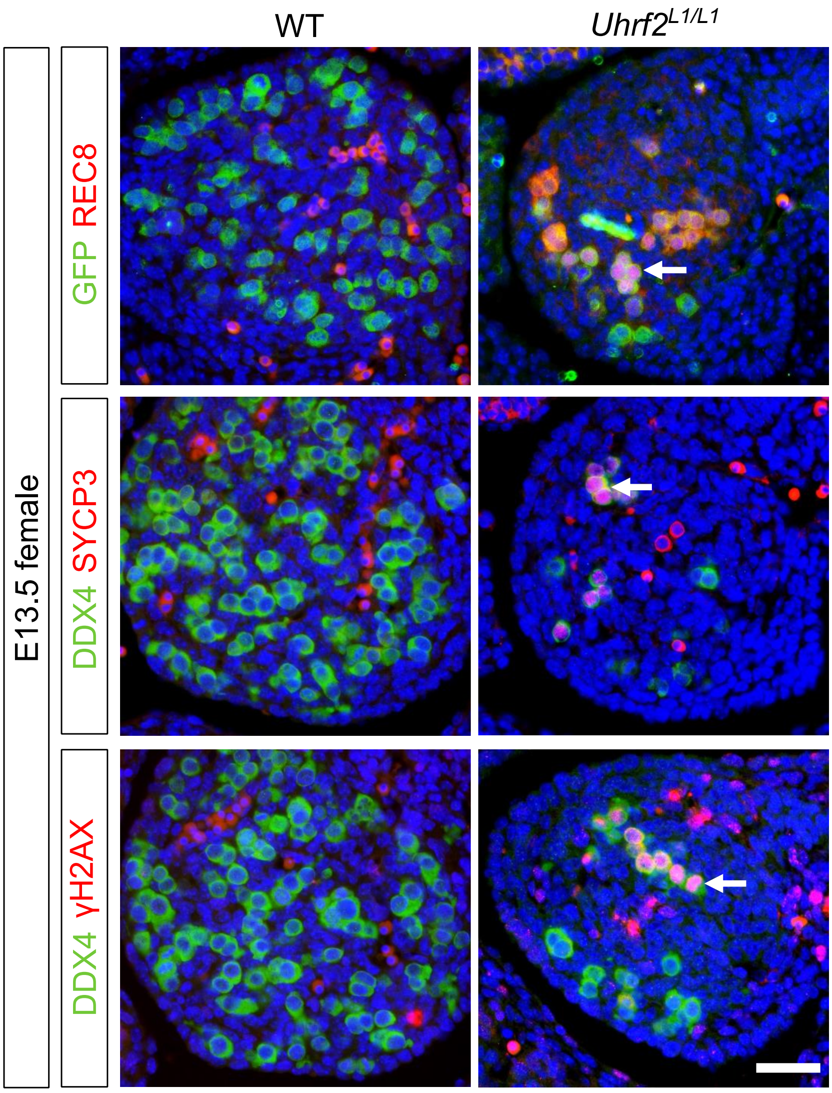

Figure S9. Markers of meiotic prophase I are detected at E13.5 in ovaries of mutants lacking UHRF2. Immunostainings of meiotic markers REC8, SYCP3 or phospho-histone γH2AX (red nuclear signals) was performed on sections of E13.5 ovaries from control (WT) and *Uhrf2*$^{L1/L1}$ fetuses, as indicated. Immunostaining of Oct4-GFP or DDX4 (green signals) was used to detect the PGCs. The white arrows point to meiotic germ cells. Nuclei are counterstained with DAPI (blue signal). The experiments were repeated independently at least on four gonads for each genotype. Scale bar: 25 µm.

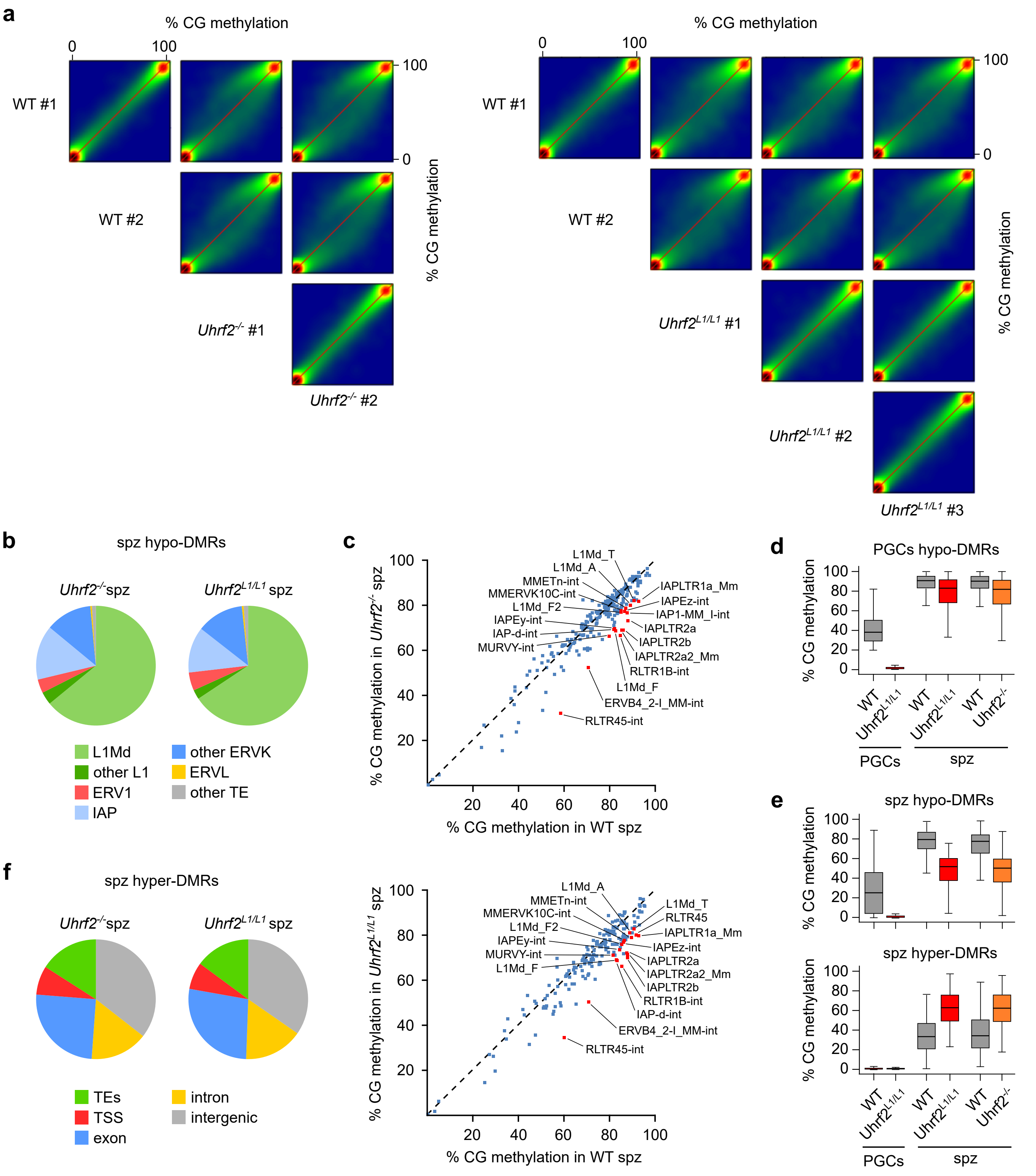

Figure S10. DNA methylation alterations in *Uhrf2*-deficient spermatozoa. a) Correlations of CG methylation scores in 500 bp windows between biological replicates of RRBS in *Uhrf2*-deficient spermatozoa compared to WT littermate controls. **b)** Proportions of transposable element families in hypomethylated DMRs from *Uhrf2*$^{-/-}$ and *Uhrf2*$^{L1/L1}$ spermatozoa. **c)** Quantification of CG methylation of transposable element families in *Uhrf2*$^{-/-}$ and *Uhrf2*$^{L1/L1}$ spermatozoa compared to their WT controls. Only families covered by > 40 CGs in WT RRBS datasets are shown. The most hypomethylated retrotransposons families are colored in red. **d)** Boxplots representing the methylation levels of *Uhrf2*$^{L1/L1}$ PGCs hypo-DMRs in PGCs and spermatozoa (n=5457 DMRs). This illustrates that a proportion of the sequences demethylated in *Uhrf2*-deficient PGCs do not regain full methylation in spermatozoa. **e)** Boxplots representing the methylation levels of *Uhrf2*$^{L1/L1}$ spermatozoa hypo-DMRs (top, n=950 DMRs) and hyper-DMRs (bottom, n=766 DMRs) in PGCs and spermatozoa. This illustrates that sperm hypo-DMRs correspond to sequences resistent to DNA demethylation in PGCs, whereas sperm hyper-DMRs are not methylated in PGCs. Boxplots: center line indicates the median, box limits indicate upper and lower quartiles, whiskers extend to 1.5 interquartile range. **f)** Proportions of genomic features in hypermethylated DMRs from *Uhrf2*$^{-/-}$ and *Uhrf2*$^{L1/L1}$ spermatozoa.
