# Supplementary material for: UHRF2 mediates resistance to DNA methylation reprogramming in primordial germ cells
Source: Nat Commun. 2025 Aug 9;16:7350. doi: 10.1038/s41467-025-61954-0 (PMC12335541; doi:10.1038/s41467-025-61954-0)
Supplement: Supplementary file 2 — Description of Additional Supplementary Files [file 41467_2025_61954_MOESM2_ESM.pdf]

Description of Additional Supplementary File

Supplementary Data 1: Sequencing statistics for the RRBS datasets generated in this study: number of reads, mapping statistics, estimated bisulfite conversion, sequencing depth and number of covered CpGs.

Supplementary Data 2: Methylation values of transposable element families (TEs) measured by RRBS during PGC development.

Supplementary Data 3: Expression analysis of genes by RNA-seq in Uhrf2 mutant and control E8.5 embryos.

Supplementary Data 4: Expression analysis of genes and transposable element families (TEs) by RNA-seq in Uhrf2 mutant and control PGCs isolated from E13.5 embryos.

Supplementary Data 5: List of primary antibodies used in immunofluorescence experiments in this study.
