# Supplementary material for: UHRF2 mediates resistance to DNA methylation reprogramming in primordial germ cells
Source: Nat Commun. 2025 Aug 9;16:7350. doi: 10.1038/s41467-025-61954-0 (PMC12335541; doi:10.1038/s41467-025-61954-0)
Supplement: Supplementary file 8 — Reporting Summary [file 41467_2025_61954_MOESM8_ESM.pdf]

nature portfolio

Corresponding author(s): Michael WEBER
Last updated by author(s): 2024/10/30

Reporting Summary

Nature Portfolio wishes to improve the reproducibility of the work that we publish. This form provides structure for consistency and transparency in reporting. For further information on Nature Portfolio policies, see our Editorial Policies and the Editorial Policy Checklist.

Statistics

For all statistical analyses, confirm that the following items are present in the figure legend, table legend, main text, or Methods section.

n/a	Confirmed	
☐	☒	The exact sample size (*n*) for each experimental group/condition, given as a discrete number and unit of measurement
☐	☒	A statement on whether measurements were taken from distinct samples or whether the same sample was measured repeatedly
☐	☒	The statistical test(s) used AND whether they are one- or two-sided<br>*Only common tests should be described solely by name; describe more complex techniques in the Methods section.*
☒	☐	A description of all covariates tested
☒	☐	A description of any assumptions or corrections, such as tests of normality and adjustment for multiple comparisons
☐	☒	A full description of the statistical parameters including central tendency (e.g. means) or other basic estimates (e.g. regression coefficient) AND variation (e.g. standard deviation) or associated estimates of uncertainty (e.g. confidence intervals)
☐	☒	For null hypothesis testing, the test statistic (e.g. *F*, *t*, *r*) with confidence intervals, effect sizes, degrees of freedom and *P* value noted<br>*Give P values as exact values whenever suitable.*
☒	☐	For Bayesian analysis, information on the choice of priors and Markov chain Monte Carlo settings
☒	☐	For hierarchical and complex designs, identification of the appropriate level for tests and full reporting of outcomes
☒	☐	Estimates of effect sizes (e.g. Cohen's *d*, Pearson's *r*), indicating how they were calculated

Our web collection on statistics for biologists contains articles on many of the points above.

Software and code

Policy information about availability of computer code

Data collection	All software and code used is open source and publicly available. Data was processed with Trim Galore v0.4.2, BSMAP v2.74, TopHat2 v2.0.13, bowtie v2-2.3.0.
Data analysis	All software and code used for data analysis is open source and publicly available. Data analysis was performed with the R software, the IRanges R package, the methylKit R package, the seqLogo R package, the RSeQC package v2.4, the Integrative Genomics Viewer (IGV) v2.8.3, HTSeq v0.9.1, DESeq2 v1.20.0, Rsubread v1.30.9, bedtools v2.27.1, bedGraphToBigWig, bwtool extract v1.0, VolcanoseR, DAVID v2021, GraphPad Prism v8.0.2, ImageJ v2.14.0.

For manuscripts utilizing custom algorithms or software that are central to the research but not yet described in published literature, software must be made available to editors and reviewers. We strongly encourage code deposition in a community repository (e.g. GitHub). See the Nature Portfolio guidelines for submitting code & software for further information.

Data

Policy information about availability of data

All manuscripts must include a data availability statement. This statement should provide the following information, where applicable:

- Accession codes, unique identifiers, or web links for publicly available datasets
- A description of any restrictions on data availability
- For clinical datasets or third party data, please ensure that the statement adheres to our policy

The sequencing datasets generated in this study (RRBS and RNA-seq) have been deposited in the NCBI Gene Expression Omnibus (GEO) under the accession number GSE240971. The following datasets were also used: RRBS in E7.5 epiblast (GSM1471900), RRBS in sperm (GSM1471911), MethylC-Seq in male and female E13.5 PGCs (GSE56697), RNA-seq in Dnmt1-cKO E13.5 PGCs (GSE74938), RNA-seq in female Setdb1-cKO E13.5 PGCs (GSE60377), RNA-seq in female Ezh2-cKO E13.5 PGCs (GSE141182), H3K9me3 and H3K27me3 ChIP-seq in E13.5 PGCs (GSE60377). Source data are provided with this paper.

Research involving human participants, their data, or biological material

Policy information about studies with human participants or human data. See also policy information about sex, gender (identity/presentation), and sexual orientation and race, ethnicity and racism.

Reporting on sex and gender	not applicable
Reporting on race, ethnicity, or other socially relevant groupings	not applicable
Population characteristics	not applicable
Recruitment	not applicable
Ethics oversight	not applicable

Note that full information on the approval of the study protocol must also be provided in the manuscript.

Field-specific reporting

Please select the one below that is the best fit for your research. If you are not sure, read the appropriate sections before making your selection.

☒ Life sciences ☐ Behavioural & social sciences ☐ Ecological, evolutionary & environmental sciences

For a reference copy of the document with all sections, see nature.com/documents/nr-reporting-summary-flat.pdf

Life sciences study design

All studies must disclose on these points even when the disclosure is negative.

Sample size	No statistical method was used to predetermine sample size. Statistical treatment was performed only on data with at least 4 independent replicates.
Data exclusions	No data were excluded from the analyses.
Replication	All experimental findings were reproduced in independent biological replicates as described in the figure legends.
Randomization	The samples were allocated into experimental groups based on their genotype. The mutant and control animals were taken from the same litters to minimize experimental variations.
Blinding	The investigators were not blinded to group allocation during data collection and analysis because the animals needed to be genotyped before performing the experiments.

Reporting for specific materials, systems and methods

We require information from authors about some types of materials, experimental systems and methods used in many studies. Here, indicate whether each material, system or method listed is relevant to your study. If you are not sure if a list item applies to your research, read the appropriate section before selecting a response.

Materials & experimental systems

n/a	Involved in the study
☐	☒ Antibodies
☐	☒ Eukaryotic cell lines
☒	☐ Palaeontology and archaeology
☐	☒ Animals and other organisms
☒	☐ Clinical data
☒	☐ Dual use research of concern
☒	☐ Plants

Methods

n/a	Involved in the study
☒	☐ ChIP-seq
☐	☒ Flow cytometry
☒	☐ MRI-based neuroimaging

Antibodies

Antibodies used	UHRF1 (H8, Santa Cruz, sc-373750); UHRF2 (C-10, Santa Cruz, sc-398953); FLAG (Origen, TA50011-100); α-TUBULIN (Sigma Aldrich, T9026); ACTIN (Sigma Aldrich, A2066); GFP (Nacalai, GF090R); GFP (Aves , GFP-1020); DDX4 (Abcam , ab13840); H2AX (Merck Millipore, 05-636); REC8 (Abcam, ab192241); STRA8 (Abcam, ab49602); SYCP3 (Abcam, ab97672)
Validation	The UHRF1 sc-373750 antibody is validated for Western Blot and Immunostaining in the mouse on the manufacturer's website, in this publication (Fig S4b) and by knock-out experiments in previous publications (ex: PMID 29180567).<br>The UHRF2 sc-398953 antibody is validated for Western Blot and Immunostaining in the mouse by knock-out experiments in this publication (Fig 2e, Fig S4) and previous publications (ex: PMID 28402695).<br>The FLAG TA50011-100 antibody is validated for Western Blot in the mouse in numerous publications listed on the manufacturer's website.<br>The α-TUBULIN T9026 antibody is validated for Western Blot in the mouse in numerous publications listed on the manufacturer's website.<br>The ACTIN A2066 antibody is validated for Western Blot in the mouse in numerous publications listed on the manufacturer's website.<br>The GFP GF090R antibody is validated for Immunostaining in the mouse in several publications listed on the manufacturer's website.<br>The GFP GFP-1020 antibody is validated for Immunostaining in the mouse in several publications listed in SiteAb (https://www.citeab.com/antibodies/575208-gfp-1020-anti-green-fluorescent-protein-antibody-gfp).<br>The DDX4 ab13840 antibody is validated for Immunostaining in the mouse on the manufacturer's website and in previous publications.<br>The H2AX 05-636 antibody is validated for Immunostaining in the mouse on the manufacturer's website and in previous publications.<br>The REC8 ab192241 antibody is validated for Immunostaining in the mouse on the manufacturer's website and in previous publications.<br>The STRA8 ab49602 antibody is validated for Immunostaining in the mouse on the manufacturer's website and in previous publications.<br>The SYCP3 ab97672 antibody is validated for Immunostaining in the mouse on the manufacturer's website and in previous publications.

Eukaryotic cell lines

Policy information about cell lines and Sex and Gender in Research

Cell line source(s)	The HEK293T cell line was obtained from ATCC (CRL-3216).
Authentication	The HEK293T cell line was not authenticated.
Mycoplasma contamination	The HEK293T cell line was tested negative for mycoplasma contamination.
Commonly misidentified lines<br>(See ICLAC register)	No commonly misidentified cell lines were used in the study

Animals and other research organisms

Policy information about studies involving animals; ARRIVE guidelines recommended for reporting animal research, and Sex and Gender in Research

Laboratory animals	We used the GOF18-Oct4ΔPE-GFP, Dnmt1<tm2Jae>,129-Alpltm1(cre)Nagy/J, C57BL/6N-Uhrf2<tm1b(EUCOMM)Wtsi> and C57BL/6N-Uhrf2<tm1c(EUCOMM)Wtsi> mouse lines. The mice were put to death to collect samples at embryonic stages (E8.5 to E17.5) or postnatally.
Wild animals	The study did not involve wild animals.
Reporting on sex	In this study, we analyzed the role of Uhrf2 in germ cells separately in female and male mice. The study shows that Uhrf2 has a greater impact on gene expression and gamete development in female compared to male mice. We provide data disaggregated for sex in the figures and the source data whenever this information has been collected and is pertinent.

Field-collected samples	The study did not involve samples collected from the field.
Ethics oversight	Animal experimental procedures complied with the ethical regulations and were approved by the Comité d'Ethique Régional en Expérimentation Animale de Strasbourg (CREMEAS) and the government authority.

Note that full information on the approval of the study protocol must also be provided in the manuscript.

Plants

Seed stocks	*Report on the source of all seed stocks or other plant material used. If applicable, state the seed stock centre and catalogue number. If plant specimens were collected from the field, describe the collection location, date and sampling procedures.*
Novel plant genotypes	*Describe the methods by which all novel plant genotypes were produced. This includes those generated by transgenic approaches, gene editing, chemical/radiation-based mutagenesis and hybridization. For transgenic lines, describe the transformation method, the number of independent lines analyzed and the generation upon which experiments were performed. For gene-edited lines, describe the editor used, the endogenous sequence targeted for editing, the targeting guide RNA sequence (if applicable) and how the editor was applied.*
Authentication	*Describe any authentication procedures for each seed stock used or novel genotype generated. Describe any experiments used to assess the effect of a mutation and, where applicable, how potential secondary effects (e.g. second site T-DNA insertions, mosiacism, off-target gene editing) were examined.*

Flow Cytometry

Plots

Confirm that:

☒ The axis labels state the marker and fluorochrome used (e.g. CD4-FITC).

☒ The axis scales are clearly visible. Include numbers along axes only for bottom left plot of group (a 'group' is an analysis of identical markers).

☐ All plots are contour plots with outliers or pseudocolor plots.

☒ A numerical value for number of cells or percentage (with statistics) is provided.

Methodology

Sample preparation	Germ cells were obtained by natural mating. The morning of the vaginal plug was designated E0.5 and all dissections were performed at 10am. The inferior part of the embryo (E9.5-E11.5) or gonads (E12.5-17.5) were dissected in PBS 1X and dissociated for 5 min at 37°C in M2 medium (Sigma-Aldrich M7167), 0.25% Trypsin, 0.16mg/mL DNAse and 1/12Vo Accumax (Millipore SCR006) to obtain a single-cell suspension. The cells were filtered on a 70µM Cell Strainer (MACS SmartStrainer, Miltenyi Biotec 130-098-462) and sorted using a FACSVantage flow cytometer (BD Biosciences) to isolate GFP-positive cells. For isolation of Uhrf2-deficient PGCs, Uhrf2-/+ and Uhrf2L1/+ females were crossed respectively with Uhrf2-/+ Oct4-GFP1/0 and Uhrf2L1/+ Oct4-GFP1/0 males and GFP-positive cells were sorted using a FACSAria Fusion cell sorter (BD Biosciences). PGCs of Dnmt1-cKO embryos were isolated using the SSEA-1 (CD15) surface marker by incubating cell suspensions 10 min at 4°C with anti-SSEA-1-PE antibodies (Miltenyi Biotec 130-104-936, 1:50) diluted in M2 medium with 4µg/mL of DNAse I and 2% FBS, followed by cell sorting using a FACSVantage flow cytometer (BD Biosciences).
Instrument	The samples were sorted using a FACSVantage SE (BD Biosciences) or a FACSAria Fusion (BD Biosciences).
Software	The samples were sorted using Cell Quest Pro 4 (FACS Vantage SE) or FACSDiva 9.0 software (FACSAria Fusion).
Cell population abundance	PGCs represent > 95 % in sorted cells. The purity of the PGCs was validated by phosphatase alkaline staining and by DNA methylation analyses.
Gating strategy	The preliminary FSC/SSC gates [FSC-A > 50 000 & SSC-A >30 000] representing total cells were further gated on singlets using first a SSC-H/SSC-W plot [50 000 > SSC-W >80 000 & SSC-H < 250 000], then a FSC-H/FSC-W plot [50 > FSC-W > 90 000 & FSC-H < 250 000]. The boundaries between positive vs negative cells were defined using a negative control cell population and based on our previous experiments.

☒ Tick this box to confirm that a figure exemplifying the gating strategy is provided in the Supplementary Information.
